# Detection of periodontal pathogens in mothers of preterm birth and/or low weight

**DOI:** 10.4317/medoral.23135

**Published:** 2019-10-27

**Authors:** Nayra Rodrigues Vasconcelos Calixto, Cláudia Maria Coêlho Alves, Luciene Maria Gomes Abreu, Erika Barbara Abreu Fonseca Thomaz, Flávia Castello Branco Vidal, Isaac Suzart Filho, Fernanda Ferreira Lopes

**Affiliations:** 1 Graduate Program in Dentistry, Federal University of Maranhão, São Luís-MA, Brazil; 2 PhD, Graduate Program of Dentistry, Federal University of Maranhão, São Luís - MA, Brazil; 3 Department of Public Health, Federal University of Maranhão, São Luís - MA, Brazil; 4 Department of Morphology, Federal University of Maranhão, São Luís - MA, Brazil; 5 Department of Health, Feira de Santana State University, Feira de Santana-BA, Brazil

## Abstract

**Background:**

The present study aims to estimate the possible relationship between periodontal pathogens in the oral cavity and the birth of Preterm Birth (PTB) and/or Low Birth Weight (LBW).

**Material and Methods:**

It’s a case- control study with the subgengival biofilm samples were collected from four sites up deeper until 48 hours postpartum and were processes by Polymerase Chain Reaction (PCR) for presence the periodontal pathogens *Prevotella intermedia (Pi), Fusobacterium nucleatum (Fn), Porphyromonas gingivalis (Pg), Treponema denticola (Td),  Tannerella forsythia (Tf) e Aggregatibacter actinomycetemcomitans (Aa)*. The mothers were divided into case grup (babies weighing < 2500g and/or gestational age < 37 weeks) and control group (babies weighing ≥ 2500g and gestational age ≥ 37 weeks). Chi-square test and the measure of association obtained by Odds Ratio (OR) were used to estimate the association between the variables.

**Results:**

Microbial analyses results showed no significant association between PTB and LBW with most periodontal pathogens in the oral cavity, even with association with the clinical presence of periodontitis.

**Conclusions:**

given the high presence of periodontal pathogens in the biofilm subgengival of recent mothers, it is suggested that the findings of this research serve as the basis for future studies on the pathophysiology involved in the relationship between periodontitis and PTB and/or LBW.

** Key words:**Low Birth Weight Infant. Premature Infant. Periodontal Diseases. Bacteria. Polymerase Chain Reaction.

## Introduction

About 15 million babies are born premature (before 37 weeks of gestation) and these babies have Low Birth Weight (LBW) (weigh less than 2,500g) (1). Preterm Birth (PTB) and Low Birth Weight (LBW) is a health problem due to the increased risk of mortality and morbidity among babies born with this condition (2), and, even with the great advances in medicine, the rate of LBW associated with PTB has not decreased along the last few decades (3). The theory of focal infection, reset recently, suggests that oral infection plays a role in systemic diseases (4). Thus, the oral infections are not limited only to the oral cavity, but oral pathogens can also induce effects adverse in other areas of the body, such as obstetric complications, such as PTB and LBW (5).

The association between periodontal disease and adverse outcomes of pregnancy is object of study for a long time (5), some authors argue that there is a significant relationship between preterm labor and gum disease (2,6), as well as studies it did not reveal adverse effects of periodontal disease in pregnant women (7). The exact mechanisms by which periodontal disease could adversely affect pregnancy still remain undefined (8). However, some studies have shown the presence of DNA of oral pathogens associated with periodontitis in amniotic fluid (9,10), placental tissues (11) and genital tract (12).

The periodontal pathogens **Prevotella intermedia* (*Pi*)*, **Fusobacterium nucleatum* (*Fn*)*, **Porphyromonas gingivalis* (Pg)*, **Treponema denticola* (*Td*)* and **Tannerella forsythia* (*Tf*)* were cited among the oral bacteria detected in extra-oral sites of women who had adverse effects in pregnancy (13). Therefore, the identification of bacterial species and subspecies may be plausible for early detection of risks to the adversities of pregnancy (9).

Even the periodontitis is considered a potential risk factor to PTB (6), contradictory data are still being published, being required more studies (1,2,6). Considering the lack of consensus in the literature on the subject, this study aimed to estimate the possible relationship between the presence of periodontal pathogens **Prevotella intermedia* (*Pi*)*, **Fusobacterium nucleatum* (*Fn*)*, **Porphyromonas gingivalis* (Pg)*, **Treponema denticola* (*Td*)*, Tannerella forsythia (*Tf*) e Aggregatibacter actinomycetemcomitans (*Aa*) in the oral cavity and the birth of PTB and/or LBW.

## Material and Methods

- Study design and sample

We conducted a case-control study of hospital based on mother-child Unit of the University Hospital of the Federal University of Maranhão, São Luís - Maranhão - Brazil. The research was approved by Ethics Committee of the University Hospital of the Federal University of Maranhão, Maranhão, Brazil (protocol nº 002673 / 2011-60). Informed consent was obtained from all individual participants included in the study.

In the sample, women were included in the age group between 12:45 years and who gave birth between October 2011 and December 2012, and remained in the institution until one week after giving birth. Were excluded mothers with cardiovascular disease and diabetes, which require antibiotic prophylaxis for dental treatment or that have been treated with periodontal treatment in pregnancy, recent mothers with HIV virus or sickle cell anemia, and mothers who made use of abortive medication.

Constituted the Group if PTB and/or LBW, mothers of children born with weight < 2500g and/or gestational age < 37 weeks. The participants in the Control Group were recent mothers who had babies with weight ≥ 2500 g and gestational age ≥ 37 weeks. All participants were examined during the same period in the two study groups.

- People and health information

A single interviewer trained collected personal information (age, education), on the condition health (hypertension, urinary tract infection), and obstetric data (PTB and LBW). Data were also obtained from the hospital records, clinical and complementary examinations records, for reliability.

- Clinical Analysis of Periodontal Condition and Biofilm Collection 

After the interview, a single examiner performed the periodontal examination, done in the bed until 48 hours postpartum, with front light and recent mothers in supine position. All 267 recent mothers, between cases and controls, were for the subgingival biofilm collection at the end of clinical periodontal examination, which was started with the Clinical Probing Depth (PCS) recorded in 6 sites for each tooth (mesiobuccal and mesio- lingual, distobuccal and disto-lingual and a measure in the midbuccal and mid lingual region). Have been recorded also the Clinical Attachment Level (CAL), Bleeding on Probing (BP), Plaque Index (PI). It was considered with periodontal disease when presented at least four teeth, with at least one site, with depth of probing ≥ 4 mm, insertion loss of 3 mm or more and bleeding on probing at the same site (14).

The subgingival biofilm was collected after removal of the supragingival plaque with sterile gaze, and then removed from four different sites (those with the largest probing depth of each quadrant) with periodontal curettes Gracey 5-6 / 7-8 (Trinity, São Paulo) inserted throughout the depth of the site removing the dental biofilm. Biofilms were packed into type eppendorfs sterile tubes containing Tris-EDTA buffer solution (150 μL Tris-EDTA + 100 μL of NaOH solution) and stored in a freezer at -20 °C.

- Bacterial Detection by Polymerase Chain Reaction (PCR)

**Figure 1:**
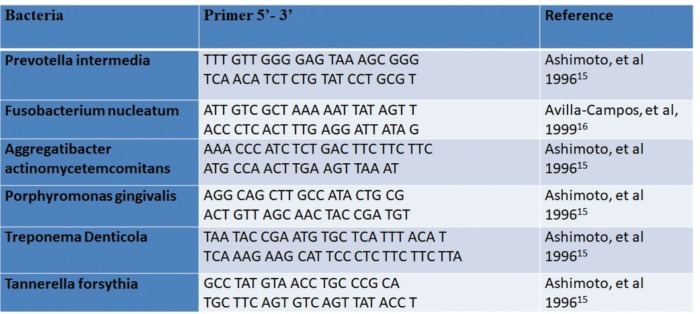
Species-specific primers that where used the reactions of PCR.

Was extracted DNA from samples as described by the QI*Aa*mp DNA usage manual (QIAGENr), with modifications. Then they were analyzed using the PCR to detect the presence of the following pathogens: *Pi*, *Fn*, A), Pg, *Td* and *Tf*. Samples of 50μL containing 45μL Mix Platinum of single bottle (Containing Taq DNA polymerase, Antobody anti-Taq planitinum, Magnesium (Mg++), DNTPs, glycerol and blue trace dyes) (Life Technologies, USA), 1.5μL primer of the bacterium under study (Invitrogen, USA) and 5μL of DNA from the sample. The primers used for each bacterial species are described in Fig. 1.

Amplification was performed on a thermal cycler (Perkin Elmer, GeneAmp PCR System 2400, Norwalk, CT, USA). The cycling used for *Pi* and *Fn* was initial denaturation at 95° for 3min; followed by 35 cycles at 95° for 1 min, 57° for 1 min and 72° for 2 min. The final denaturation was 72° for 7 min (19). For the bacteria *Tf*, Pg and *Td* the cycling was initial denaturation at 94° for 3 min; followed by 40 cycles at 94° for 30s, 62° for 30s and at 72° for 30s. The final denaturation was 72° for 5min (10). For bacteria *Aa* the cycling used was initial denaturation at 95° for 3min; followed by 35 cycles at 95° for 30s, 55° for 30s and at 72° for 1min. The final denaturation was 72° for 7min (10).

The amplification products were visualized in agarose gel electrophoresis to 1.5% in TBE buffer (Tris 1M, 0.9M boric acid, EDTA 0.01M, pH 8) (Life Technologies, USA), stained with gel loading (Life Technologies, USA) and Gel red (Uniscience, USA) and photographed under a UV light transilluminator (BIO-RAD Laboratories, USA), the 100 bp molecular weight standard (Uniscience, USA) was included.

- Statistical analysis

The dependent variable was the occurrence of gestational adverse outcomes (PTB and LBW) and the independent variables included the presence of factors related to the mother's profile and periodontal pathogens. Descriptive statistics were performed and categorical variables were compared between the groups using the Chi-square test and the association measure obtained by the Odds Ratio (OR), with their respective 95% confidence intervals (95% CI) to estimate the association between the presence of periodontal pathogens and the gestational outcomes. The level of significance was 5% (*p*<0.05).

## Results

The present study included 261 mothers in the municipality of São Luís, MA, Brazil, with ages varying from 12 to 43 years (mean: 26.04 ± 6.77 years). The sample consisted of 175 mothers in the control group and 86 in the case group. The case and control groups were similar as: age of the mother, number of children and presence of urinary infection. Lower schooling, presence of hypertension and periodontitis were significantly more common in the case group (Table 1).

The probability of having periodontitis or hypertension among postpartum women who had PTB and / or low weight infants is 4 times higher for both factors, both of which are considered risk factors with strong association. Lower maternal schooling may also be considered a laughing factor for the study outcome, through a weak association (Table 2).

The results of microbial analyzes showed no significant association between PTB and LBW and most of the periodontal pathogens under study, except for *Tannerella forsythia* and Aggregatibacter actinomycetemcomitans (Table 3). The Table 4 shows that the lower limit of the confidence interval for all six periodontal pathogens studied was less than 1 and could not be considered a risk factor for the study outcome.

**Table 1:**
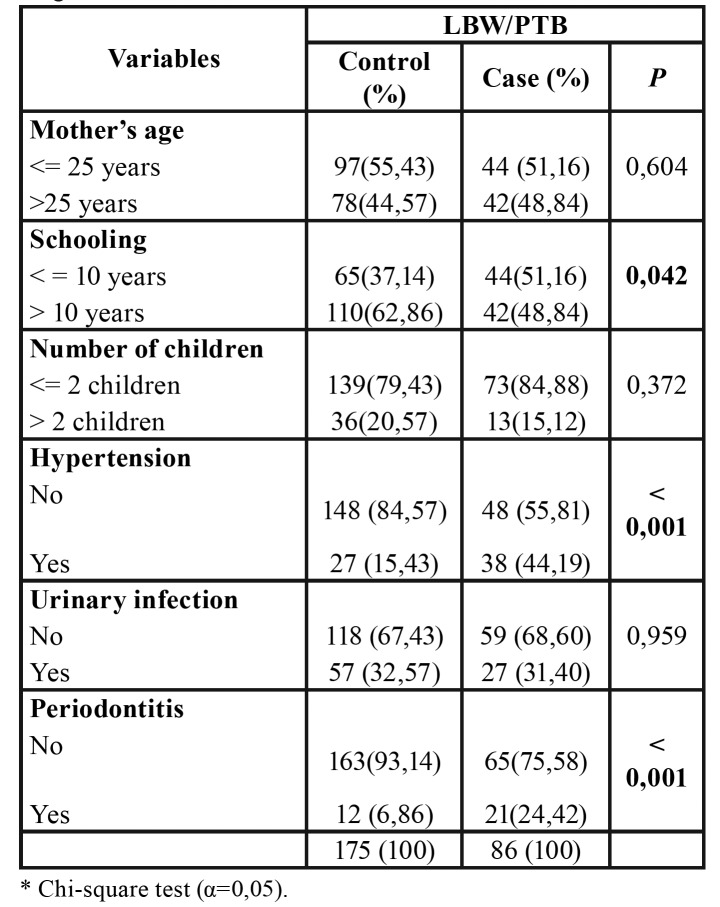
Characteristics of the sample consisting of mothers without and with the gestational outcome preterm birth and / or low birth weight.

**Table 2:**
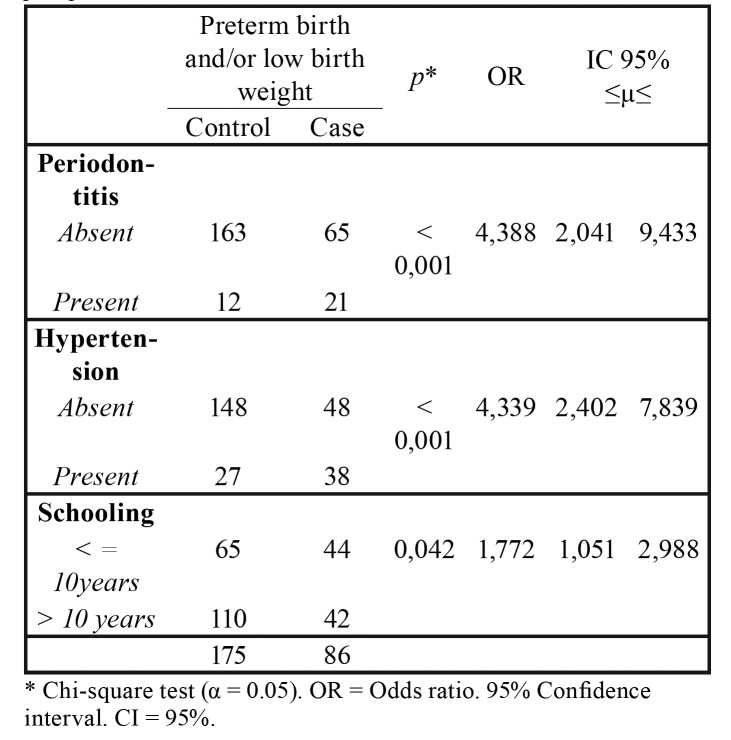
Measurement of association between preterm birth and / or low birth weight with periodontitis, hypertension and schooling in puerperal women.

**Table 3:**
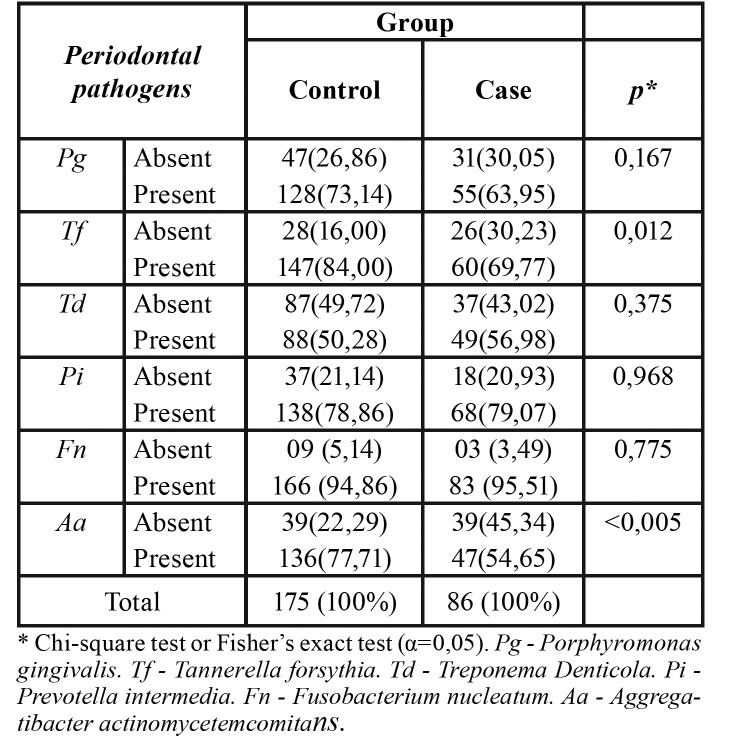
Association between preterm birth and / or low birth weight and periodontal pathogens Pg, *Tf*, *Td*, *Pi*, *Fn*, *Aa* detected in mothers.

**Table 4:**
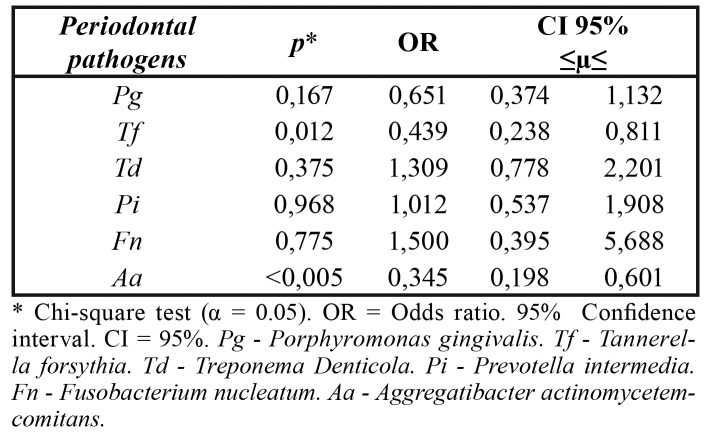
Measure of association between preterm birth and / or low birth weight with the periodontal pathogens detected in puerperal women

## Discussion

The present study showed that periodontitis can be considered a risk factor to the birth of PTB and/or LBW, However, the results of microbial analyses showed no significant relationship between the periodontal pathogens detected in recent mothers and PTB and/or LBW.

The recent mothers of PTB and/or low weight show increased frequency of periodontitis and hypertension, with significant differences in comparative analysis between control and the outcome assessed for both exposure variables (*p*<0.001). Highlights that the association was strong for periodontitis (OR=4.388) and for high blood pressure (OR=4,339), suggesting to be a causal risk factor. The literature mentions that hypertension is an acclaimed influencer factor associated with the outcome of PTB and LBW (17), while, periodontitis has been considered potential risk factor to PTB (6), with from OR magnitude from 2.09 to 4.19 (2).

Although there are studies that found significant relationship between preterm labor and gum disease (2,18), there is evidence of the absence of adverse effects of periodontal disease in pregnant women (19). One of the factors that can explain this divergence of results is the fact that there is no universally applied standard for diagnosis of periodontitis (2).

High frequency was observed all periodontal pathogens analyzed in recent mothers, revealing similar occurrence rate between the case and control groups, except for the periodontal pathogens *Tf* and *Aa*. This result corroborates the already found previously, even with greater occurrence of *Aa* in recent mothers of babies to term (20). A possible explanation for these findings can be in the event that certain groups of individuals are more prone to colonization of *Aa* (21).

The high frequency of *Pi* periodontal pathogens and *Fn* in the Subgingival biofilm analyzed. Result that can be explained because higher proportion of "red" and "orange" bacterial complex were encountered in subgingival specimens (22). The *Fn* has been detected in a wide variety of placental and fetal tissues including amniotic fluid, fetal membranes, umbilical cord blood, gastric aspirates, lungs and neonatal fetal stomach, associated with chorioamnionitis, preeclampsia, PTB and neonatal sepsis (23). The prevalence of *Fn* detected in the blood of the umbilical cord of neonatal sepsis is equal or superior to *E. coli* e Streptococcus in Group B, putting *Fn* on the same scale of importance that these two pathogens neonatal diseases well-recognized (23). Highlight a case report of fetal death caused by *Fn* oral, because this is the first human evidence that the bacteria originated from subgingival plaque of the mother can translocate to the placenta and the fetus, causing acute inflammation and causing the fetus to death (24). The *Fn* is affected by environmental factors, being one of the most abundant species of bacteria from oral cavity in both healthy and unhealthy individuals (25), which could be proven with the findings of the present research.

Although there is a chance that the Pg may be related to adverse pregnancy outcomes, including PTB and LBW (26), the results of this research have failed to establish this relationship. However, the great breadth of confidence registered for periodontal pathogens Pg, *Td*, *Pi* and *Fn*, these factors lead to exposure from the classification of the protection factor risk factor to the outcome under study, suggesting that other factors can be influenced, such as the response of the host, which was not investigated in this study.

There is the new model of the pathogenesis by which periodontal disease is initiated by a microbial community synergy and dysbiotic and not "periopatógenos" selected, which converge to form and stabilize a provocative microbiota of diseases (27). We highlight that this research has detected the presence of pathogens, without determining the level and your proportion in both study groups. However, the discussion about causality/casualty associated with periodontal disease is gaining space in the scientific community in recent years (28), which makes the results of this study relevant. Thus, it is still important to define whether the relationship between periodontitis and PTB and LBW babies is casual or if there is a simple association (20).

The research investigated 06 (six) periodontal pathogens, and more than 300 bacterial species may be present in oral cavity colonization (29), so periodontal disease is not caused only by the bacteria studied in this study. Another important aspect that may be able to explain the results of this study is that during pregnancy levels of these pathogens are increased, due to their greater ease of proliferation (25). Corroborating this reasoning, higher levels of pathogens of periodontal pathogens were found in pregnant women when they compared the count of 10 different bacterial species extracted from the subgingival biofilm of pregnant and non-pregnant women (28). Therefore, it is important to investigate the fact that periodontal pathogens have a relationship with PTB and/or LBW babies, since maternal periodontal infection may directly and/or indirectly have the potential to influence the health of the maternal-fetal unit (30).

The research presents several strengths as a robust diagnostic criterion of periodontitis, sample size respecting the case/control relationship and the precision of the microbial detection technique of periodontal pathogens. Thus, it can be concluded that there is a relationship between PTB and/or LBW infants and periodontitis, suggesting that this oral pathology may be considered a risk factor for gestational adversities, however, it was not possible to attribute causality by means of the presence of the bacteria under study. However, given the high presence of periodontal pathogens in the subgingival biofilm of the mothers, it is suggested that the findings of this research serve as a basis for future studies on the pathophysiology involved in the relationship between periodontitis and PTB and LBW infants.
